# Is the main goal of mastication achieved after orthodontic treatment? A prospective longitudinal study

**DOI:** 10.1590/2177-6709.22.3.072-078.oar

**Published:** 2017

**Authors:** Gustavo Hauber Gameiro, Isabela Brandão Magalhães, Mariana Marcon Szymanski, Annicele Silva Andrade

**Affiliations:** 1 Universidade Federal do Rio Grande do Sul, Department of Physiology (Porto Alegre/RS, Brazil).; 2 Universidade Vale do Rio Verde, Department of Clinical Dentistry (Três Corações/MG, Brazil).; 3 Universidade Federal do Rio Grande do Sul, Clinical Hospital of Porto Alegre, (Porto Alegre/RS, Brazil).

**Keywords:** Malocclusion, Masticatory performance, Deglutition.

## Abstract

**Objective:**

To investigate the masticatory and swallowing performances in patients with malocclusions before and after orthodontic treatment, comparing them to an age- and gender-matched control group with normal occlusion.

**Methods:**

Twenty-three patients with malocclusions requiring orthodontic treatment were included in this prospective study. One month after appliance removal, seventeen patients completed a follow-up examination and the data were compared with those of a control group with thirty subjects with normal occlusion. Masticatory performance was determined by the median particle size for the Optocal Plus^®^ test food after 15 chewing strokes, and three variables related to swallowing were assessed: a) time and b) number of strokes needed to prepare the test-food for swallowing, and c) median particle size of the crushed particles at the moment of swallowing.

**Results:**

At the baseline examination, the malocclusion group had a significantly lower masticatory performance and did not reach the particle size reduction at the moment of swallowing, when compared with the control group. After treatment, the masticatory performance significantly improved in the malocclusion group and the particle size reduction at swallowing reached the same level as in the control group.

**Conclusions:**

The present results showed that the correction of malocclusions with fixed appliances can objectively provide positive effects in both mastication and deglutition processes, reinforcing that besides aesthetic reasons, there are also functional indications for orthodontic treatment.

## RESUMO

**Objetivo:**

investigar as performances mastigatória e de deglutição em pacientes com má oclusão, antes e após o tratamento ortodôntico, comparando-os com um grupo controle com oclusão normal, equiparado por idade e sexo.

**Métodos:**

vinte e três pacientes com má oclusão requerendo tratamento ortodôntico foram incluídos neste estudo prospectivo. Um mês após a remoção dos aparelhos, dezessete pacientes completaram os exames de acompanhamento, e seus dados foram comparados com aqueles de um grupo controle com trinta indivíduos com oclusão normal. A performance mastigatória foi determinada pelo tamanho mediano das partículas do alimento teste Optocal Plus^®^ após 15 ciclos mastigatórios, e três variáveis relacionadas à deglutição foram avaliadas: a) tempo e b) número de ciclos necessários para preparar o alimento teste para deglutição, e c) tamanho mediano das partículas trituradas no momento da deglutição.

**Resultados:**

no exame inicial, o grupo com má oclusão apresentou uma menor performance mastigatória e não atingiu a redução do tamanho das partículas no momento da deglutição, quando comparado com o grupo controle. Após o tratamento, a performance mastigatória aumentou significativamente no grupo com má oclusão, e a redução do tamanho das partículas no momento da deglutição atingiu o mesmo nível do grupo controle.

**Conclusões:**

os resultados demonstraram que a correção das más oclusões com aparelhos fixos pode, objetivamente, proporcionar efeitos positivos nos processos de mastigação e deglutição, reforçando que, além de razões estéticas, existem também indicações funcionais para o tratamento ortodôntico.

## INTRODUCTION

Currently, the aesthetic characteristics of malocclusions are the main factor in determining orthodontic treatment demand.^1^ Orthodontists are increasing the attention to the patient’s limits of soft tissue adaptation and soft tissue contours.^2^ However, the achievement of an appropriate masticatory function at the end of orthodontic treatment is another very important objective that cannot be overlooked. In fact, the need for treating malocclusions in order to achieve functional benefits is still a question under debate. Some studies reported that although the orthodontic and/or surgical correction of malocclusions usually improves the self-estimated masticatory ability (the individual’s own assessment of mastication), the masticatory performance (the objective measurement of mastication) is still impaired in these patients compared to subjects with normal occlusion.^3^ ^,^ ^4^ On the other hand, some studies have shown that orthodontic treatment is able to restore the masticatory performance and also other variables related to mastication, such as occlusal force and occlusal contacts.^5^ ^,^ ^6^

Most of the studies are cross-sectional, and few reports have objectively compared the masticatory function in the same patient between pre- and post-treatment. Moreover, a wide-range search of the literature was unable to find a prospective study in which the swallowing function of orthodontics patients has been evaluated. The urge to swallow food could be triggered by a threshold level in the food particle size as well as by degree of lubrication of the food bolus.^7^ Usually, individuals with a significantly impaired masticatory performance (eg. incomplete dentition) try to compensate their deficiencies by chewing longer, but this cannot prevent them from swallowing larger food particles.^8^ An acceptable particle size distribution of the food bolus before swallowing is considered as a crucial criterion in the assessment of normality of the masticatory function, because a lack of reduction in ingested food particles can increase the gastric emptying rate^9^ ^,^ ^10^ and increase the susceptibility to some gastric diseases.^11^ ^,^ ^12^

The present study was designed to investigate the masticatory and swallowing performances in patients before and after the orthodontic treatment, comparing them to a control group with normal occlusion.

## MATERIAL AND METHODS

The orthodontic treatment group comprised twenty three consecutive patients selected from private practice offices after an initial screening examination. Seventeen (9 men and 8 women, aged 24.2 ± 6 years) patients completed the follow-up records after the treatment and the following inclusion criteria were considered: an uneventful medical history and good oral health; an approximately equal number of occlusal units (an occluding molar pair is counted as two occlusal units, whereas a premolar pair is counted as one occlusal unit)^13^ with malocclusions requiring orthodontic treatment. Ten individuals had Class I malocclusions, five had Class II, and three had Class III. All patients were treated with full fixed appliances (Roth prescription, slot 0.022-in) and all cases were treated without extractions. The exclusion criteria were: previous orthodontic treatment or symptoms of temporomandibular joint dysfunction. Thirty volunteers aged 20 ± 5 years with normal occlusion were selected from the students of Faculty of Dentistry of Federal University of Rio Grande do Sul to comprise the control group. The occlusion was considered normal if the following criteria were met: Class I canine and molar relationships with minor or no crowding, normal overjet and overbite (1-3 mm) and normal transversal occlusion. The patients and the control group were homogeneous with regard to sex, age and facial type. The facial type was established by radiographic (patients group) or photographic evaluation (control group). Informed written consent was obtained from all participants prior to their enrollment in the study. The Ethics Committees of Federal University of Rio Grande do Sul approved the protocol. Sample size was determined based on clinically relevant masticatory performance data from the literature,^11^ ^,^ ^14^ with a power of 80% and α?#8197;= 0.05. Ultimately, twenty individuals were deemed ideal for this prospective longitudinal study. The research started with twenty three patients, but six did not return to follow-up examination. All variables of the study were registered before and after treatment in the patients group, with the interval of approximately three years between the two examinations, and the control group was evaluated once. 

The masticatory performance was evaluated by the individual capacity of fragmentation of an artificial test-food (Optocal).^15^ Subjects were given 17 cubes (3.0 g) and instructed to chew for 15 cycles, which were monitored visually by a trained examiner and timed using a digital stopwatch. After the 15 strokes, the subjects spat the particles into a plastic cup, rinsed their mouth with water and spat the remaining mouth contents until all particles were removed. The particles were washed and dried for 24 h in a stove at 60^o^C. After that, they were sieved through a stack of up to 10 sieves, with square apertures decreasing from 5.6 to 0.5 mm, for 5 min. Median particle sizes (X50) were determined as previously described.^16^The X50 is defined as the aperture of a theoretical sieve through which 50% of the weight can pass. Deterioration in masticatory performance will lead to an increase in the X50.

The swallowing function was evaluated by three different variables: a) time and b) number of strokes needed to prepare the food for swallowing, and c) the median particle size (X50) of the crushed particles that were expectorated just before a subject felt the need to swallow.^17^ For this study, the participants received one set of Optocal cubes and were instructed to chew until they felt the urge to swallow. A trained examiner counted the number of chewing cycles and registered the total time of the cycles (in seconds), which was measured with a digital chronometer. When the patient reported the urge to swallow, the test was stopped and the participants were asked to spit the comminuted particles into a plastic cup containing a 50 µm paper filter. The swallowing threshold particles were submitted to the same fragment size analysis as was done for the masticatory performance test, aforementioned. Therefore, a small X50 meant better masticatory performance and swallowing threshold.

Shapiro-Wilks tests were used to verify data normality, and variables were not normally distributed. For intra-group comparisons (before vs. after treatment), variables were analyzed by Wilcoxon test, and the Mann-Whitney test was used for the intergroup comparisons. The relationships between the median particle sizes and the other masticatory or swallowing variables were evaluated by Spearman rank correlation analysis. SPSS software version 19.0 was used for all analyses, and the significance level was set at *p*< 0.05.

The X50 data of 10 subjects in the control group were also analyzed with the Dahlberg formula EM = √∑d^2^/2n and paired *t*-tests after two analyses with a 7-day interval. There was no statistical difference between the evaluations (*p*> 0.05), and the reproducibility error was less than 10% for the X50 (0.5 mm).

## RESULTS

The results of masticatory variables registered during the masticatory performance tests are shown in Table 1. Before treatment, the median particle size after 15 chewing strokes (X50-15) was higher in the patients than in the control group (5.7 mm*vs.*4.8 mm, *p*< 0.05). After treatment, the masticatory performance had significantly improved in the patients group and reached similar levels (5.1 mm) as in the control group, as confirmed by the absence of significant differences between groups. The total time spent during mastication and the time of each masticatory cycle did not change after treatment in the patients group. The inter-group differences were also not significant regarding these variables.

**Table 1:** Mean/standard deviation of the median particle size chewed for 15 cycles (X50, in mm), total chewing time and duration of each cycle during the masticatory performance test before and after orthodontic treatment.

Variables	Malocclusion group Before treatment	Malocclusion group After treatment	Control group (normal occlusion)
Masticatory performance (X50-15)	5,7^a^*/0,6	5,1^b^/0,9	4,8/1,1
Total chewing time (sec.)	10,9^a^/2,0	11,8ª/1,9	11,9/2,7
Duration of each chewing cycle (sec.)	0,7^a^ /0,1	0,8^a^/0,2	0,8/0,2

Distinct letters indicate statistical difference between periods in the patients group (Wilcoxon test, *p* < 0.05).

* indicates statistical differences in relation to the control group (Mann-Whitney test, *p*< 0.05).

The data regarding swallowing variables are shown in Table 2. The swallowing thresholds (time and number of strokes) for Optocal did not vary significantly after treatment in the patients group. These variables registered both before and after treatment were also similar to those observed in the control group. However, the median particle size of the particles at the moment of swallowing (X50-sw) was significantly affected by the orthodontic treatment. Before treatment, the X50-sw was higher in the patients than in the control group (4.5 mm *vs.* 3.0 mm, *p*< 0.05). After treatment, the X50-sw had significantly decreased in the patients group and reached similar levels (3.4 mm) as in the control group, indicating an improvement in the swallowing threshold of patients.

**Table 2:** Mean/standard deviation of the median particle size (X50, in mm) before swallowing and swallowing thresholds (number of strokes and total time)registered before and after orthodontic treatment.

Variables	Malocclusion group Before treatment	Malocclusion group After treatment	Control group (normal occlusion)
Median particle size (X50-sw)	4,5^a^*/1,5	3,4^b^/0,7	3,0/1,1
Total chewing time (sec.)	28,2^a^/11,3	27,3ª/7,6	24,6/9,9
Number of chewing cycles (N)	39^a^/13	38^a^/12	35/15

Distinct letters indicate statistical difference between periods in the patients group (Wilcoxon test, *p*< 0.05).

* indicates statistical differences in relation to the control group (Mann-Whitney test, *p*< 0.05).

Table 3 shows the correlations between the X50 of the particles at the moment of swallowing (X50-sw), the X50 of the particles after 15 chewing strokes (X50-15) and the other swallowing thresholds. Significant positive correlations between the X50 of swallowing (X50-sw) and the X50 after 15 strokes (X50-15) were observed in the control group, and also in the patients group after treatment. In this group, these variables were not significantly correlated before treatment. The X50 of swallowing (X50-sw) were negatively correlated with the other swallowing variables (number of strokes and total time) in both the patients (before and after treatment) and in the control group. Therefore, if subjects used more strokes before swallowing, the collected particles were smaller, indicating a better swallowing threshold.

**Table 3:** Spearman’s Correlation Coefficients (r) and p-value regarding the median particle size at the swallowing moment (X50-sw), the median particle size after 15 masticatory cycles (X50-15) and the other swallowing variables in the malocclusion (before and after treatment) and control groups.

			X50-masticatory performance	Total time until swallowing (sec.)	Number of chewing cycles used before swallowing (N)
Patients Before treatment	X50 (sw)	p	0.87	0.04	0.007
		r	-0.03	-0.39*	-0.50*
	X50 (15)	P	-	0.10	0.22
		r	-	0.32	0.23
Patients After treatment	X50 (sw)	p	0.04	0.04	0.02
		r	0.55*	-0.59*	-0.63*
	X50 (15)	p	-	0.62	0.10
		r	-	0.09	0.32
Control Group	X50 (sw)	p	0.02	0.007	0.006
		r	0.40*	-0.48*	-0.58*
	X50 (15)	p	-	0.32	0.13
		r	-	0.10	0.28

*p< 0.05 (Spearman’s correlation).

## DISCUSSION

There have been several reports on the functional benefits induced by orthodontic treatment in clinical practice^18^ ^-^ ^20^ However, to the best of our knowledge, the present study is the first to longitudinally examine the effects of orthodontic treatment on both mastication and swallowing performances using only objective measures. These objective analyses are necessary considering that patients normally overestimate their masticatory capacity when they are evaluated only by subjective methods. For example, many patients with compromised dentition or dentures report they possess a good masticatory capacity, even when objective tests show values ​​much lower than in subjects with a natural dentition.^21^ The present study shows that patients with malocclusions have a decreased masticatory performance when compared to individuals with normal occlusion. This impaired masticatory function also reflects on the swallowing performance of these patients, in which the particle size reduction at time of swallowing was significantly poorer than for their same-age normal occlusion counterparts. One month after treatment, however, the patients exhibited similar results to those of the control group in both the masticatory and swallowing tests (Tables 1 and 2). 

Previous studies reported similar findings. The self-masticatory ability of girls with Class II was significantly improved after orthodontic treatment, but the masticatory performance values did not reach those in the normal occlusion group.^3^ Another study has demonstrated that the masticatory performance of patients submitted to orthognathic surgery for mandibular advancement has significantly improved 5 years after treatment, although the values were still impaired compared with controls.^22^ These studies cannot be directly compared to the present one, because the samples were very different regarding the severity of the malocclusion and also the age of the groups. The first study evaluated adolescents during active stages of dental and craniofacial development. This could be a confounding factor since the masticatory performance naturally improves during these stages.^3^ In the second study, the age of the patients was similar to the present research, but the severity of the malocclusions wasn’t. Moreover, these previous studies did not take into account the vertical facial types of the patients. It has been reported that the facial patterns can influence the masticatory performance indices.^23^ Therefore, care to control this possible confounding factor in the comparison of patients *vs.* controls was taken into account in the present study.

The results of masticatory performance tests show that patients with malocclusions were not able to appropriately chew or swallow before the orthodontic treatment (Table 1). This finding is in line with several studies that have reported a significant relationship between malocclusions and decreased masticatory performance.^24^ ^-^ ^26^ Reduced muscle activity and a reduced occlusal contact area are probably the main factors responsible for this decreased masticatory performance of patients with malocclusions.^26^ It is well known that adequate occlusal contacts promote mandibular stability at maximal intercuspation and have a significant influence on chewing function and masticatory muscle activity.^27^ ^,^ ^28^

Interestingly, the decreased masticatory performance of patients before treatment had a significant influence in the swallowing performances. The present results show that patients before treatment exhibit similar values to the control group regarding all the swallowing variables, in exception for the median particle size at the moment of swallowing, the X50-sw (Table 2). These findings demonstrate that patients with malocclusions did not compensate their impaired masticatory performance by increasing the number of strokes to the first swallowing. Therefore, they will swallow larger food particles, as evidenced by the larger X50-sw observed in this group. After treatment, the particle size reduction at time of swallowing was significantly improved in the patients group and reached similar level as in the control group. The other swallowing variables remained unchanged after treatment, indicating a real improvement in the swallowing performance, because patients were able to pulverize the test food better than before treatment without increasing the number of chewing strokes.

Patients with malocclusions could in theory increase the number of chewing strokes they make before swallowing so that they swallow food particles of the same size as individuals with normal occlusion. However, this fact did not occur. The median particle sizes obtained after 15 strokes did not correlate with the number of strokes used before swallowing, both in the patients and in the control groups (Table 3). That is to say, bad chewers did not increase the number of cycles before swallowing. Probably, patients with malocclusions are unaware of their reduced masticatory capacity and thus swallow larger food particles. This finding is in accordance with a previous study, in which subjects with malocclusions did not swallow their food after a greater number of chewing strokes than subjects with normal occlusion.^29^ Taken together, these results show that the ability of subjects with malocclusions to reach the goal of mastication (to prepare a food bolus that is safe to swallow) could be outside the normal physiological range. The establishment of a precise bolus particle size before swallowing is triggered represents an important requirement to the healthy functioning of the gastrointestinal system. A dysfunctional deglutition is known to be linked to high morbidity.^30^ Moreover, an impaired reduction of ingested food particles is not only able to increase the gastric emptying rate,^9^ ^,^ ^10^ but also could be related with more severe chronic inflammatory changes and *Helicobacter pylori* infection of the gastric mucosa, especially in the antrum of the stomach.^11^

Significant positive correlations between the median particle size after 15 strokes and the particle size at the moment of swallowing were found both in the control group and in the patients group after treatment (Table 3). This result suggests that orthodontic treatment could have increase the oral sensitivity regarding the particle size distribution of ready-to-swallow food boluses. The main explanation for this improvement is probably the increased number of occlusal contacts achieved after treatment. Although this variable has not been evaluated in the present study, previous studies have shown that orthodontic treatment can increase the occlusal contact area and occlusal force,^5^ ^,^ ^6^ which are important factors in the determination of both sensorial and motor aspects of mastication.^21^ ^,^ ^26^

One limitation of the present study is that our sample comprised different types of malocclusions. Considering that factors such as occlusal contacts and the type and severity of malocclusion have a significant impact on masticatory performance,^26^ ^-^ ^29^ the evaluation of these factors in subjects with specific malocclusions would give us a broader view about the topic. However, this limitation does not reduce the merit of the present results. It is important to point out that significant differences were observed for almost all variables in the patients group when appropriate statistical intragroup comparisons were evaluated. The inclusion of a control group with homogeneous characteristics in relation to possible confounding factors (eg. sex, age, facial pattern, number of occlusal units) also strengthens the findings of the manuscript. A future study should include patients with specific types of malocclusions in order to characterize possible significant differences regarding the impact of orthodontic treatment in their masticatory and swallowing performances.

## CONCLUSIONS

» Patients with malocclusions present impaired masticatory and swallowing functions, since their food bolus contain much larger particles than the normal occlusion counterparts.
» At long-term follow-up examination, masticatory and swallowing performances are reestablished to those observed in the normal occlusion group.

## References

[1] Mandall NA, Wright J, Conboy FM, O'Brien KD (2001). The relationship between normative orthodontic treatment need and measures of consumer perception. Community Dent Health.

[2] Proffit WR (2000). The soft tissue paradigm in orthodontic diagnosis and treatment planning a new view for a new century. J Esthet Dent.

[3] Henrikson T, Ekberg E, Nilner M (2009). Can orthodontic treatment improve mastication A controlled, prospective and longitudinal study. Swed Dent J.

[4] van der Bilt A, Engelen L, Pereira LJ, van der Glas HW, Abbink JH (2006). Oral physiology and mastication. Physiol Behav.

[5] Makino E, Nomura M, Motegi E, Iijima Y, Ishii T, Koizumi Y (2014). Effect of orthodontic treatment on occlusal condition and masticatory function. Bull Tokyo Dent Coll.

[6] Aszkler RM, Preston CB, Saltaji H, Tabbaa S (2014). Long-term occlusal changes assessed by the American Board of Orthodontics' model grading system. Am J Orthod Dentofacial Orthop.

[7] Prinz JF, Lucas PW (1995). Swallow thresholds in human mastication. Arch Oral Biol.

[8] Mishellany-Dutour A, Renaud J, Peyron MA, Rimek F, Woda A (2008). Is the goal of mastication reached in young dentates, aged dentates and aged denture wearers. Br J Nutr.

[9] Holt S, Reid J, Taylor TV, Tothill P, Heading RC (1982). Gastric emptying of solids in man. Gut.

[10] Pera P, Bucca C, Borro P, Bernocco C, De LA, Carossa S (2002). Influence of mastication on gastric emptying. J Dent Res.

[11] Sierpinska T, Golebiewska M, Dlugosz J, Kemona A, Laszewicz W (2007). Connection between masticatory efficiency and pathomorphologic changes in gastric mucosa. Quintessence Int.

[12] Carretero D, Sánchez-Ayala A, Rodriguez A, Lagravère MO, Gonçalves TM, Garcia RC (2011). Relationship between non-ulcerative functional dyspepsia, occlusal pairs and masticatory performance in partially edentulous elderly persons. Gerodontology.

[13] Kayser AF (1981). Shortened dental arches and oral function. J Oral Rehabil.

[14] Fontijn-Tekamp FA, Slagter AP, Van Der Bilt A, Van 'T Hof MA, Witter DJ, Kalk W (2000). Biting and chewing in overdentures, full dentures, and natural dentitions. J Dent Res.

[15] Pocztaruk RL, Frasca LC, Rivaldo EG, Fernandes EL, Gavião MB (2008). Protocol for production of a chewable material for masticatory function tests (Optocal - Brazilian version). Braz Oral Res.

[16] van der Bilt A, van der Glas HW, Mowlana F, Heath MR (1993). A comparison between sieving and optical scanning for the determination of particle size distributions obtained by mastication in man. Arch Oral Biol.

[17] Fontijn-Tekamp FA, van der Bilt A, Abbink JH, Bosman F (2004). Swallowing threshold and masticatory performance in dentate adults. Physiol Behav.

[18] Bergius M, Kiliaridis S, Berggren U (2000). Pain in orthodontics A review and discussion of the literature. J Orofac Orthop.

[19] Brown DF, Moerenhout RG (1991). The pain experience and psychological adjustment to orthodontic treatment of preadolescents, adolescents, and adults. Am J Orthod Dentofacial Orthop.

[20] Erdinç AM, Dinçer B (2004). Perception of pain during orthodontic treatment with fixed appliances. Eur J Orthod.

[21] Van der Bilt A (2011). Assessment of mastication with implications for oral rehabilitation a review. J Oral Rehabil.

[22] van den Braber W, van der Bilt A, van der Glas H, Rosenberg T, Koole R (2006). The influence of mandibular advancement surgery on oral function in retrognathic patients a 5-year follow-up study. J Oral Maxillofac Surg.

[23] Gomes SG, Custodio W, Faot F, Del Bel Cury AA, Garcia RC (2010). Masticatory features, EMG activity and muscle effort of subjects with different facial patterns. J Oral Rehabil.

[24] Henrikson T, Ekberg EC, Nilner M (1998). Masticatory efficiency and ability in relation to occlusion and mandibular dysfunction in girls. Int J Prosthodont.

[25] van den Braber W, van der Glas HW, van der Bilt A, Bosman F (2001). Chewing efficiency of pre-orthognathic surgery patients selection and breakage of food particles. Eur J Oral Sci.

[26] Magalhães IB, Pereira LJ, Marques LS, Gameiro GH (2010). The influence of malocclusion on masticatory performance. A systematic review. Angle Orthod.

[27] Owens S, Buschang PH, Throckmorton GS, Palmer L, English J (2002). Masticatory performance and areas of occlusal contact and near contact in subjects with normal occlusion and malocclusion. Am J Orthod Dentofacial Orthop.

[28] Ferrario VF, Serrao G, Dellavia C, Caruso E, Sforza C (2002). Relationship between the number of occlusal contacts and masticatory muscle activity in healthy young adults. Cranio.

[29] English JD, Buschang PH, Throckmorton GS (2002). Does malocclusion affect masticatory performance. Angle Orthod.

[30] Danesh-Sani SA, Rahimdoost A, Soltani M, Ghiyasi M, Haghdoost N, Sabzali-Zanjankhah S (2013). Clinical assessment of orofacial manifestations in 500 patients with multiple sclerosis. J Oral Maxillofac Surg.

